# Association of Habitual Physical Activity With the Risk of All-Cause Mortality Among Chinese Adults: A Prospective Cohort Study

**DOI:** 10.3389/fpubh.2022.919306

**Published:** 2022-06-24

**Authors:** Peng Hu, Murui Zheng, Jun Huang, Wenjing Zhao, Harry H. X. Wang, Xiong Zhang, Yuanyuan Chen, Hai Deng, Pengzhe Qin, Xudong Liu

**Affiliations:** ^1^Department of Epidemiology, School of Public Health, Sun Yat-sen University, Guangzhou, China; ^2^Department of Epidemiology and Health Statistics, School of Public Health, Guangdong Pharmaceutical University, Guangzhou, China; ^3^Department of Community Health, Guangzhou Center for Disease Control and Prevention, Guangzhou, China; ^4^Department of Geriatrics, Institute of Geriatrics, Guangdong Provincial People's Hospital, Guangdong Academy of Medical Science, Guangzhou, China; ^5^School of Public Health and Emergency Management, Southern University of Science and Technology, Shenzhen, China; ^6^Faculty of Medicine, JC School of Public Health and Primary Care, The Chinese University of Hong Kong, Hong Kong, Hong Kong SAR, China; ^7^Department of Neurology, Maoming People's Hospital, Maoming, China; ^8^Department of Chronic Noncommunicable Disease Prevention and Control, Guangzhou Center for Disease Control and Prevention, Guangzhou, China; ^9^Department of Cardiology, Guangdong Cardiovascular Institute, Guangdong Provincial People's Hospital, Guangdong Academy of Medical Science, Guangzhou, China

**Keywords:** habitual physical activity, leisure-time physical activity, commute activity, mortality, cohort study

## Abstract

**Objective:**

This study was conducted to evaluate the association of the risk of all-cause mortality with habitual physical activity (HPA) and its different domains among Chinese adults.

**Methods:**

A total of 11,994 participants from the Guangzhou Heart Study were followed up until 1 January 2020. Information on HPA, including leisure-time physical activity (LTPA) and commute activity, was collected using a modified Global Physical Activity Questionnaire. Individual cause of death was obtained from the National Death Registry of China. Cox proportional hazards regression model was used to estimate hazard ratio (*HR*) and 95% confidence interval (*CI*) after adjustment for covariates.

**Results:**

During 37,715 person-years of follow-up, 208 deaths (1.73%) were observed. When compared with the highest with the lowest exposure tertiles, HPA and LTPA were associated with 34% (*HR*: 0.66, 95% *CI*: 0.46–0.95) and 30% (*HR*: 0.70, 95% *CI*: 0.49–0.99) reduced risk of all-cause mortality after adjustment for covariates. Commute activity was not associated with mortality risk. For the specific component of LTPA, we found that every 1 MET-h/week increment of the housework was associated with a 1% (*HR*: 0.99, 95% *CI*: 0.98–0.99) decreased mortality risk, and performing brisk walking/health exercises/Yangko was associated with a 46% reduced mortality risk (*HR*: 0.54, 95% *CI*: 0.29–0.99).

**Conclusion:**

This study suggests that a higher level of HPA and LTPA was associated with a lower risk of all-cause mortality. Our findings suggest people to perform HPA, especially LTPA, as a strategy for mortality reduction and health promotion.

## Introduction

Physical activity is one of the most important modifiable lifestyle factors affecting health and longevity (1, 2). Increasing epidemiological studies evidenced a protective role of a higher level of physical activity in the long run in reducing the risk of all-cause and specific mortality (3–7). Physical activity guidelines from the World Health Organization (WHO), America, and China recommended that at least 150 min of moderate-intensity or 75 min vigorous-intensity aerobic physical activity per week, or an equivalent combination of both should be performed for substantial health benefit (8–10).

Individuals' preference for habitual physical activity (HPA) varies greatly for the different social classes and cultural backgrounds (11, 12); hence, it is necessary to explore the effects of different kinds of physical activity on health. A systematic review and meta-analysis included 48 studies showed that compared with the recommended level of physical activity, higher physical activity was associated with lower risk of all-cause and cardiovascular disease mortality (13). Several studies suggested that regular leisure-time physical activity (LTPA) may be associated with a decreased risk of all-cause mortality (3, 4, 14–16). However, few studies in China concentrated on the specific LTPA. An active commute is gradually regarded as an important source of physical activity in recent years (17, 18). A previous meta-analysis with 829,098 workers indicated that active commuting could decrease mainly all-cause and cardiovascular mortality with a dose–response relationship (17). A recent study also found that compared with the car used, physically active commuting modes were associated with a range of health benefits (18); however, other studies did not find such an association between commute activity and mortality (19, 20).

Therefore, in this current study, we included 11,994 middle-aged older Chinese adults to prospectively examine the association of all-cause mortality with HPA and its different domains among Chinese adults.

## Methods

### Study Population

The participants in this study were recruited from the Guangzhou Heart Study (GZHS), an ongoing community-based prospective cohort study. The details of the cohort can be seen in our previous reports (21–23). In brief, a total of 12,013 permanent residents aged ≥35 years were recruited by using the multistage sampling method, and a baseline survey was accomplished between July 2015 and August 2017. The inclusion criteria for the GZHS were permanent residents who had a Guangzhou household register, had lived in the selected communities for at least 6 months before the survey, and aged 35 years or above were included (21). Those who had mental or cognitive disorders, had mobility difficulties, and had malignant tumors under treatments were excluded; those who were pregnant or lactating women, and were non-Guangzhou permanent residents were excluded (21). In this study, 19 participants who had incomplete information on covariates were also excluded.

Finally, a total of 11,994 participants from GZHS were included for further analysis. This study was approved by the Ethical Review Committee for Biomedical Research, School of Public Health, Sun Yat-Sen University, and was conducted adhering to the Declaration of Helsinki.

### Outcome of Mortality

The individual cause of death up to 1 January 2020 was obtained from the National Death Registry of China in Guangzhou Center for Disease Control and Prevention. The follow-up period in our study was defined as the time from participation in the cohort to the death date for decedents or to the censoring date for survivors. The causes of death were coded by professional medical workers according to the 10th revision of the International Classification of Disease.

### Habitual Physical Activity Evaluation

The HPA was assessed at baseline survey using the modified Global Physical Activity Questionnaire (GPAQ) by using in-person interview (24). The Chinese version of GPAQ was found to have a good validation and reliability (25). Two dimensions of habitual physical activity, including LTPA and commute activity, were evaluated according to the method in our previous report (22). Participants were asked to provide information of the average frequency and duration of each kind of LTPA and commute activity. A metabolic equivalent (MET, 1 MET = 1 kcal/h/kg) was used to indicate the intensity of each physical activity (26). According to the guidelines on physical activity by WHO, to attain substantial health benefits, adults should perform at least 150 min of moderate-intensity or 75 min vigorous-intensity aerobic physical activity per week, or an equivalent combination of both should be performed (9); this means that conducting activity with at least 10 MET-h/week is suggested to reach the minimum level of the recommended standard (9).

For LTPA, the information of the eight most common LTPA categories, such as Tai Chi/Qigong, housework, stroll, bicycling, brisk walking/exercises/Yangko, swimming, ball games (basketball, table tennis, badminton, etc.), long-distance running/aerobics dancing, were investigated (22). Then a value of 3.0 METs, 3.2 METs, 3.5 METs, 3.5 METs, 5.3 METs, 6.0 METs, 6.0 METs, and 6.5 METs was assigned, respectively, to each of above eight common kinds of LTPA orderly according to the Ainsworth's Compendium of Physical Activity (26). When any other specific type of LTPA was mentioned, it would be grouped into one of the above corresponding categories in line with the specific content.

For commute activity, the eight most common commutes including riding a bicycle, riding a motorcycle, self-driving, taking a bus, taking the subway, boating, walking, and working at home were investigated (22). Then a value of 6.8 METs, 3.5 METs, 2.5 METs, 1.3 METs, 1.3 METs, 1.3 METs, 3.5 METs, and 1.3 METs was assigned to each of the above eight commutes orderly according to the Ainsworth's compendium of physical activities (26).

The volume of each specific physical activity was calculated by multiplying the duration of activity by its frequency and then by its intensity, and the Met-h per week (MET-h/week) was used to describe the volume of each specific physical activity. The total volume of HPA for each participant was calculated as the sum of the volume of each LTPA and each commute activity.

### Potential Confounders

A face-to-face approach was adopted for the baseline survey. Social demographics, personal lifestyle, and medical history were collected by using a structured questionnaire (22, 23). Height, weight, and blood pressure were performed by using the standard instruments according to protocols (21). Body mass index (BMI, kg/m^2^) was calculated as weight divided by height squared. Hypertension was defined as systolic blood pressure ≥140 mmHg or diastolic blood pressure ≥90 mmHg, or self-reported physician-diagnosed hypertension (22). A fasting blood sample was taken in the morning to detect the serum concentrations of blood glucose and lipid. Diabetes (27) was defined as fasting blood glucose ≥7.0 mmol/L or self-reported physician-diagnosed diabetes. Dyslipidemia (28) was defined as serum cholesterol ≥ 5.2 mmol/L, low-density lipoprotein cholesterol ≥ 3.4 mmol/L, triglyceride ≥ 1.7 mmol/L, or self-reported physician-diagnosed dyslipidemia.

The following covariables from the baseline survey were considered in the analysis: age (years), sex (male or female), education (lower than high school, high school, and college or above), marital status (married, others), body mass index (kg/m^2^), smoking (never, former, and current), alcohol drinking (never, occasionally, and frequent), hypertension (yes or no), diabetes (yes or no), dyslipidemia (yes or no), physician-diagnosed chronic obstructive pulmonary disease (COPD, yes or no), and physician-diagnosed cardiovascular diseases (yes or no).

### Statistical Analysis

A chi-square test for categorical variables and a *t*-test or one-way analysis of variance for continuous variables were used to compare the distribution difference. The tertile method was used to transform the continuous variable of HPA and LTPA into categorical variables. The continuous variable of commute activity was then transformed into categorical variables as retirement, low (<10 MET-h/week), and high (≥10 MET-h/week). Schoenfeld residuals were used to examine the proportional hazard assumption in the crude model and adjusted model, respectively, and the results satisfied the assumption. Crude and adjusted hazard ratios (*HRs*) with their 95% confidence intervals (*CIs*) were estimated by using the Cox proportional hazard regression model. The linear exposure–response relationship was examined by putting the median of each tertile of exposure as a continuous variable into the model. The potential non-linear relationship was examined using restricted cubic spline regression (knots on the 50th, 75th, and 95th percentiles), with *HR* and 95% *CI* being calculated based on the Cox regression model. The restricted cubic spline regression was performed with the “rms” package.

Several sensitivity analyses were conducted. To test the robustness of the results, we further categorize the HPA and LTPA as ≤ 10 MET-h/week, 10–50 MET-h/week, >50 MET-h/week, corresponding to <150 min per week, 150–750 min per week, and more than 750 min per week of moderate-intensity physical activity. To exclude the possibility of reverse causality, we conducted the analysis by excluding participants who died within the first year after they were recruited. To exclude the influence of age-related factors, we performed the analysis by excluding participants aged 80 years or above. To eliminate the impact of accidents, we repeated the analysis by excluding participants who died due to traffic accidents and injuries. All statistical analyses were performed using R 4.0.1 (R Development Core Team, Vienna, Austria); the tests were two-tailed, and the *p*-value of <0.05 was considered statistically significant.

## Results

**Figure 1:**
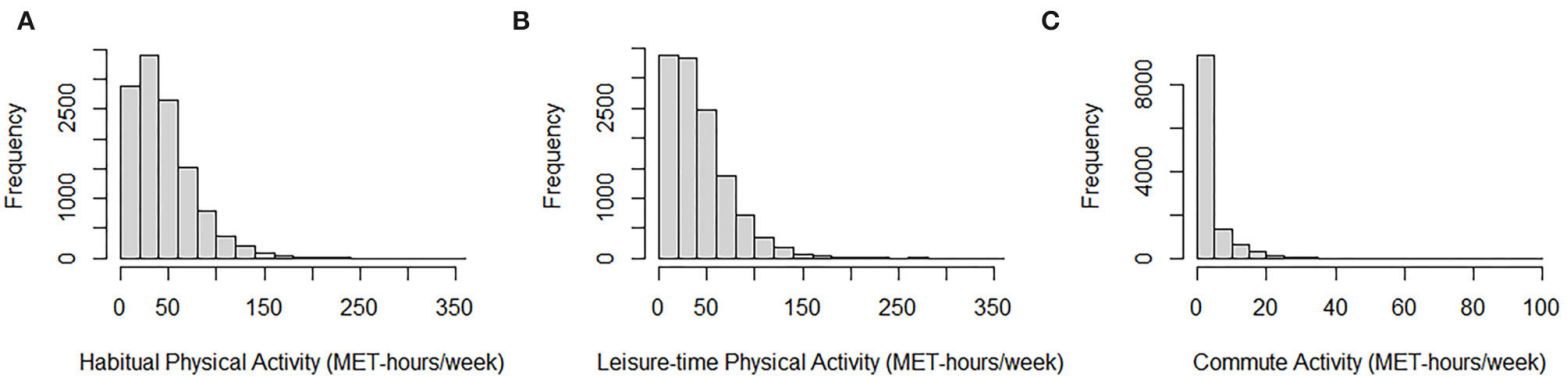
Histograms of habitual physical activity, leisure-time physical activity, and commute activity. **(A)** Histogram of habitual physical activity among all participants. **(B)** Histogram of leisure-time physical activity among all participants. **(C)** Histogram of commute activity among all participants.

The distribution of HPA, LTPA, and commute activity were shown in Figure 1. A total of 11,994 participants were included, and 208 deaths (1.73%) were observed during the follow-up. As shown in Table 1, of all participants, the mean (S.D.) of age and BMI was 58.67 (11.78) years and 23.98 (3.53) kg/m^2^. The median (interquartile) of the follow-up period was 3.27 (0.64) years, and the median (interquartile) of HPA, LTPA, and commute activity was 37.10 (39.58) MET-h/week, 34.65 (41.65) MET-h/week, and 0.00 (2.92) MET-h/week. Most participants were women (65.03%), non-smokers (78.69%), and never drinkers (78.66%). Participants who died were more likely to have a history of cardiovascular diseases, COPD, hypertension, regular alcohol consumption, and current smoking (*p* < 0.05). The detailed distribution of these characteristics across tertiles of HPA volume was shown in Supplementary Table S1.

**Table 1:** The characteristics of participants.

**Characteristic**	**Total** **(*N* = 11994)**	**Lived** **(*N* = 11786)**	**Death** **(*N* = 208)**	***P-*value**
Age, years, mean (S.D.)	58.67 (11.78)	58.45 (11.68)	71.08 (10.79)	<0.001^*^
Body mass index, kg/m^2^, mean (S.D.)	23.98 (3.527)	23.99 (3.522)	23.28 (3.770)	0.007^*^
Follow-up period, years, median (interquartile)	3.27 (0.64)	3.27 (0.63)	1.80 (1.29)	<0.001^‡^
HPA, MET-h/week, median (interquartile)	37.10 (39.58)	37.43 (39.56)	26.88 (33.28)	<0.001^‡^
LTPA, MET-h/week, median (interquartile)	34.65 (41.65)	34.65 (41.50)	24.50 (34.65)	<0.001^‡^
Commute activity, MET-h/week, median (interquartile)	0.00 (2.92)	0.00 (3.13)	0.00 (0.00)	<0.001^‡^
Sex, *N* (%)				<0.001^†^
Male	4194 (34.97)	4081 (34.63)	113 (54.33)	
Female	7800 (65.03)	7705 (65.37)	95 (45.67)	
Education, *N* (%)				<0.001^†^
< high school	7518 (62.68)	7363 (62.47)	155 (74.52)	
High school	2914 (24.30)	2883 (24.46)	31 (14.90)	
> high school	1562 (13.02)	1540 (13.07)	22 (10.58)	
Marital status*, N* (%)				<0.001^†^
Married	10202 (85.10)	10054 (85.30)	148 (71.20)	
Others	1792 (14.90)	1732 (14.70)	60 (28.80)	
Smoking				<0.001^†^
Non-Smoker	9438 (78.69)	9301 (78.92)	137 (65.87)	
Ex-smoker	718 (5.99)	688 (5.84)	30 (14.42)	
Current Smoker	1838 (15.32)	1797 (15.25)	41 (19.71)	
Alcohol drinking				<0.001^†^
Never	9435 (78.66)	9258 (78.55)	177 (85.10)	
Occasion	1835 (15.30)	1817 (15.42)	18 (8.65)	
Frequent	724 (6.04)	711 (6.03)	13 (6.25)	
Hypertension				<0.001^†^
Yes	3796 (31.65)	3706 (31.44)	90 (43.27)	
No	8198 (68.35)	8080 (68.56)	118 (56.73)	
Dyslipidemia				0.804^†^
Yes	8426 (70.25)	8282 (70.27)	144 (69.23)	
No	3568 (29.75)	3504 (29.73)	64 (30.77)	
Diabetes				0.376^†^
Yes	982 (8.19)	961 (8.15)	21 (10.10)	
No	11012 (91.81)	10825 (91.85)	187 (89.90)	
COPD				<0.001^†^
Yes	674 (5.62)	644 (5.46)	30 (14.42)	
No	11320 (94.38)	11142 (94.54)	178 (85.58)	
Cardiovascular disease				<0.001^†^
Yes	599 (4.99)	575 (4.88)	24 (11.54)	
No	11395 (95.01)	11211 (95.12)	184 (88.46)	
Habitual physical activity				<0.001^†^
Tertile 1 ( ≤ 24.5 MET-h/week)	4020 (33.50)	3920 (33.30)	100 (48.10)	
Tertile 2 (>24.5– ≤ 51.1 MET-h/week)	3976 (33.10)	3911 (33.20)	65 (31.20)	
Tertile 3 (> 51.1 MET-h/week)	3998 (33.30)	3955 (33.60)	43 (20.70)	
Leisure-time physical activity				0.011^†^
Tertile 1 (≤ 22.4 MET-h/week)	4113 (34.30)	4027 (34.20)	86 (41.30)	
Tertile 2 (>22.4– ≤ 46.9 MET-h/week)	3935 (32.80)	3862 (32.80)	73 (35.10)	
Tertile 3 (> 46.9 MET-h/week)	3946 (32.90)	3897 (33.10)	49 (23.60)	
Commute activity				<0.001^†^
Retirement (0 MET-h/week)	6706 (55.90)	6544 (55.50)	162 (77.90)	
Low (<10 MET-h/week)	4019 (33.50)	3986 (33.80)	33 (15.90)	
High (≥ 10 MET-h/week)	1269 (10.60)	1256 (10.70)	13 (6.20)	

*HPA, habitual physical activity; LTPA, leisure-time physical activity; MET-h, metabolic equivalent values-hours*.

* *P-values of continuous variables were from t-test*.

† *P-values of categorical variables were from chi-square tests*.

‡ *P values from Wilcoxon rank sum test*.

As shown in Table 2, a higher level of HPA was associated with a lower risk of all-cause mortality after adjusting for confounders (*p*_lineartrend_ = 0.027); in comparison to the participants within the lowest tertile of HPA volume, those within the median tertile and the highest tertile were associated with 11% (*HR*: 0.89, 95% *CI*: 0.65–1.22) and 34% (*HR*: 0.66, 95% *CI*: 0.46–0.95) reduced risk of death; every 10 MET-h/week increments of HPA volume was associated with 5% (*HR*: 0.95, 95% *CI*: 0.90–0.99) reduced risk of death.

**Table 2:** Associations of all-cause mortality with habitual physical activity.

	**Deaths/Person-years**	Crude HR (95% CI)^*^	**Adjusted HR (95% CI)^**†**^**
Tertile 1	100 / 12533	1.00	1.00
Tertile 2	65 / 12508	0.65 (0.48, 0.89)	0.89 (0.65, 1.22)
Tertile 3	43 / 12674	0.42 (0.30, 0.61)	0.66 (0.46, 0.95)
*P* for trend		<0.001	0.027
Every 10 MET-h/week increment		0.89 (0.85, 0.94)	0.95 (0.90, 0.99)

* *Crude HR, without any adjustment*.

† *Adjusted HR, adjustment for age, sex, education, marital status, smoking, alcohol drinking, body mass index, physician-diagnosed hypertension, diabetes, dyslipidemia, cardiovascular disease, and chronic obstructive pulmonary disease*.

As shown in Table 3, a higher level of LTPA was associated with a lower risk of all-cause mortality after considering potential covariates (*p*_lineartrend_ = 0.047); in comparison to the participants within the lowest tertile of LTPA volume, those within the highest tertiles were associated with a 30% (*HR*: 0.70, 95% *CI*: 0.49–0.99) reduced risk of death; every 10 MET-h/week increments of LTPA volume was associated with a 6% reduced risk of death (*HR*: 0.94, 95% *CI*: 0.90–0.99). Commute activity was not significantly associated with the mortality risk.

**Table 3:** Associations of all-cause mortality with leisure-time physical activity and commute physical activity.

	**Deaths/Person-years**	Crude HR (95% CI)^*^	**Adjusted HR (95% CI)^**†**^**	**Adjusted HR (95% CI)^**‡**^**
**Leisure-time physical activity**				
Tertile 1	86/12835	1.00	1.00	1.00
Tertile 2	73/12385	0.88 (0.64, 1.20)	0.85 (0.62, 1.16)	0.85 (0.62, 1.17)
Tertile 3	49/12496	0.58 (0.41, 0.83)	0.68 (0.48, 0.98)	0.70 (0.49, 0.99)
*P* for trend		0.002	0.037	0.047
Every 10 MET-h/week increment		0.91 (0.87, 0.96)	0.94 (0.90, 0.99)	0.94 (0.90, 0.99)
**Commute activity**				
Retirement	162/21209	1.00	1.00	1.00
Low	33/12470	0.35 (0.24, 0.51)	1.33 (0.88, 2.01)	1.23 (0.81, 1.88)
High	13/4036	0.42 (0.24, 0.74)	1.86 (1.00, 3.42)	1.70 (0.92, 3.16)
*P* for trend		<0.001	0.040	0.086
Every 10 MET-h/week increment		0.56 (0.40, 0.80)	1.18 (0.93, 1.48)	1.15 (0.90, 1.46)

* *Without any adjustment*.

† *Adjustment for age, sex, education, marital status, smoking, alcohol drinking, body mass index, physician-diagnosed hypertension, diabetes, dyslipidemia, cardiovascular disease, and chronic obstructive pulmonary disease*.

‡ *Further adjustment for commute activity or leisure-time physical activity*.

For the specific LTPA, every 1 MET-h/week increment of the housework was associated with a 1% (*HR*: 0.99, 95% *CI*: 0.98–0.99) decreased risk of all-cause mortality after adjustment for possible covariables (Table 4); performing brisk walking/health exercises/Yangko was associated with a 46% (*HR*: 0.54, 95% *CI*: 0.29–0.99) reduced risk of all-cause mortality after considering covariables (Table 5). No significant association was found in any other types of LTPA.

**Table 4:** The association between every 1 MET-hour/week increment of specific leisure-time physical activity and the all-cause mortality risk.

	**Lived (N** **=** **11786)**		**Death (N** **=** **208)**		P value^*^		**HR (95% CI)**	
	**Mean (SD)**	**Median (IQR)**	**Mean (SD)**	**Median (IQR)**			**Crude^**†**^**	**Adjusted^**‡**^**
Stroll	11.41 (13.76)	7.00 (24.50)	14.01 (16.05)	12.25 (24.50)	0.012		1.01 (1.00, 1.02)	1.00 (0.99, 1.01)
Tai chi/Qigong	0.54 (3.35)	0.00 (0.00)	1.04 (5.71)	0.00 (0.00)	0.366		1.01 (0.98, 1.05)	0.99 (0.95, 1.03)
Brisk walking/health exercises/Yangko	2.20 (8.99)	0.00 (0.00)	1.34 (6.13)	0.00 (0.00)	0.061		0.98 (0.96, 1.00)	0.98 (0.96, 1.00)
Long-distance running/ aerobics dancing	1.57 (7.89)	0.00 (0.00)	0.66 (5.82)	0.00 (0.00)	0.011		0.98 (0.95, 1.01)	0.99 (0.97, 1.02)
Swimming	0.54 (3.79)	0.00 (0.00)	0.23 (2.42)	0.00 (0.00)	0.213		0.96 (0.90, 1.03)	0.98 (0.92, 1.04)
Ball games	1.07 (6.37)	0.00 (0.00)	0.68 (4.95)	0.00 (0.00)	0.070		0.99 (0.96, 1.02)	0.99 (0.97, 1.02)
Bicycling	0.98 (5.01)	0.00 (0.00)	0.77 (4.03)	0.00 (0.00)	0.303		0.99 (0.96, 1.02)	0.99 (0.96, 1.02)
Housework	22.19 (26.53)	11.20 (32.53)	14.12 (19.22)	10.40 (22.40)	<0.001		0.98 (0.97, 0.99)	0.99 (0.98, 0.99)
Others	1.27 (9.81)	0.00 (0.00)	0.71 (7.32)	0.00 (0.00)	0.078		0.99 (0.97, 1.02)	0.99 (0.97, 1.01)

*IQR, interquartile range; LTPA, leisure-time physical activity; MET, metabolic equivalent values; SD, standard deviation*.

* *P value for Wilcoxon rank sum test*.

† *Crude HR, without any adjustment*.

‡ *Adjustment for age, sex, education, marital status, smoking, alcohol drinking, body mass index, community activity, physician-diagnosed hypertension, diabetes, dyslipidemia, cardiovascular disease, and chronic obstructive pulmonary disease*.

**Table 5:** The association between specific leisure-time physical activity and the all-cause mortality risk.

**Specific leisure-time physical activity**	Yes^*^(Deaths/Person-years)	No^*^(Deaths/Person-years)	**Crude HR (95% CI)^**†**^**	**Adjusted HR (95% CI)^**‡**^**
Stroll	144/23083	64/14632	1.42 (1.06, 1.91)	1.12 (0.83, 1.51)
Tai chi/Qigong	11/1574	197/36141	1.27 (0.69, 2.33)	0.93 (0.51, 1.72)
Brisk walking/health exercises/Yangko	11/3470	197/34245	0.55 (0.30, 1.01)	0.54 (0.29, 0.99)
Long-distance running/aerobics dancing	4/2365	204/35350	0.29 (0.11, 0.79)	0.52 (0.19, 1.42)
Swimming	4/1381	204/36334	0.51 (0.19, 1.37)	0.74 (0.27, 2.01)
Ball games	5/2043	203/35672	0.43 (0.18, 1.03)	0.80 (0.32, 1.96)
Bicycling	12/2936	196/34779	0.72 (0.40, 1.30)	0.89 (0.49, 1.61)
Housework	122/28459	86/9256	0.46 (0.35, 0.61)	0.75 (0.56, 1.00)
Others	3/1391	205/36324	0.38 (0.12, 1.21)	0.38 (0.12, 1.20)

* *Yes means exposure to specific leisure-time physical activity (LTPA). No means not exposure to specific LTPA*.

† *Crude HR, without any adjustment*.

‡ *Adjusted HR, adjustment for age, sex, education, marital status, smoking, alcohol drinking, body mass index, community activity, physician-diagnosed hypertension, diabetes, dyslipidemia, cardiovascular disease, chronic obstructive pulmonary disease, and commute activity*.

**Figure 2:**
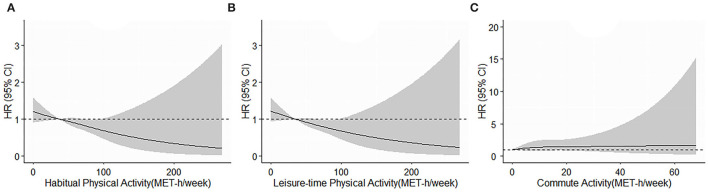
**(A–C)** Restricted cubic splines of the relationship of all all-cause mortality with habitual physical activity, leisure-time physical activity, and commute activity. Potential non-linear relationships were examined using restricted cubic splines (knots on the 50th, 75th, and 95th percentiles), with hazard ratios (*HRs*) based on cox regression models. HRs with 95% confidence intervals (*CIs*) were plotted for each unit of the exposure against the median of the first tertile. The dash line represents the *HR* equal to 1. The *HRs* was adjusted for age, sex, education, marital status, smoking, alcohol drinking, body mass index, hypertension, diabetes, dyslipidemia, chronic obstructive pulmonary disease, cardiovascular disease, leisure-time physical activity (for commute activity), and commute activity (for leisure-time physical activity).

The non-linear associations of all-cause mortality with HPA, LTPA, and commute activity were displayed in Figure 2, but no significant association was observed. In the sensitivity analysis by categorizing the HPA or LTPA as ≤ 10 MET-h/week, 10–50 MET-h/week, and >50 MET-h/week, similar results were obtained (Supplementary Table S2). The sensitivity analyses were also conducted by excluding 49 participants who died within the first year, excluding 511 participants who were 80 years or above, and excluding 9 participants who died due to traffic accidents and injuries, a similar protective effect was observed for both HPA and LTPA (Supplementary Tables S3–S5).

## Discussion

This prospective community-based cohort study displayed that a higher level of HPA and LTPA was associated with a lower risk of all-cause mortality, while no significant association was observed for the commute activity.

In this study, we found that both HPA and LTPA were related to a decreased risk of death among Chinese, with a dose–response trend. Our results were consistent with the previous reports (29, 30). A prospective cohort study with participants from 17 countries reported that a higher level of physical activity was associated with a lower risk of mortality (29). A pooled analysis with six studies from the NCI Cohort Consortium showed similar protective effects and that the maximum longevity benefit was obtained from those meeting the recommended guidelines by either moderate or vigorous-intensity activities (30). Additionally, several studies among Chinese revealed a similar protective effect between physical activity and the risk of mortality (14–16).

Consistent with the previous studies (19, 20), our study did not find the association of commute activity with all-cause mortality risk. However, a meta-analysis including 829,098 workers found that active commuting to work had a protective effect against all-cause mortality and cardiovascular mortality, with a dose–response relationship (17). Patterson et al. also indicated that compared with car use, physically active commute modes including train use and cycling were beneficial to health and associated with the decreased risk of all-cause mortality, cardiovascular disease mortality, and cancer mortality (18). This contradiction may result from differences in the accessibility of private vehicles and the quality of public transport services in different countries and regions. Another possible reason is that only a small number of subjects in our study performed active commute activity. In our study, 5.79 and 3.98% of the population performed walking and cycling, whereas 23.54% of participants chose public transportation (bus, metro, and boat) or motorized vehicles (car or motorcycle).

Our findings showed that every 1 MET-h/week increment of the housework was associated with a decreased risk of all-cause mortality, which was consistent with the previous studies (31–33). In addition, our results were like previous studies (34–36), which found that performing brisk walking/health exercises/Yangko was associated with a lower risk of mortality. However, this present study did not find a significant association between the other specific LTPA and all-cause mortality, while some previous studies showed that swimming, running, bicycling, and soccer were associated with a lower risk of mortality (35, 36). The possible reason may be that people with different social and cultural backgrounds have different preferences and participation in various leisure activities. Another possible explanation is that the limited mortality events during a relatively short-time follow-up period may contribute to wide *CIs* and the non-significant *HRs*.

The mechanism of physical activity in promoting health is complex. Physical activity might have a protective effect on enhancing the production of anti-inflammatory markers and inhibiting the production of inflammatory markers, which could help limit the development of many chronic diseases, such as cardiovascular disease and diabetes (37, 38), and ultimately reduce mortality and prolong life expectancy (39, 40). In addition, physical activity has been evidenced to improve lipid lipoprotein profiles, improve endothelial functions, and reduce bless pressure and coagulation, which is beneficial for cardiovascular health and can reduce mortality risk (41).

This study has several strengths. First, participants from community-dwelling residents were recruited by using a multi-stage sampling method (21, 22), which can minimize or reduce selection bias to a large degree. Furthermore, we conducted the questionnaire survey face to face with trained investigators, which can help to reduce the information bias. Second, we evaluated the effect of various types of activity (HPA, LTPA and its specific type, and community activity) on the mortality risk, providing relatively comprehensive evidence. Last, this study adopted the prospective design and potential confounders were collected as much as possible.

There are also several limitations. First, we measured physical activity by using the Global Physical Activity Questionnaire rather than objective measurement methods; however, adequate reliability and sufficient validity had been evidenced in many previous studies by using such investigation tools (25, 42). Second, the follow-up duration in our study was relatively short, resulting in a few death cases; however, our study was derived from an ongoing cohort and a long-term follow-up study will be conducted to verify the obtained results. Third, we did not collect the information on the duration and frequency of occupational activity, so the occupational activity cannot be quantified in this study. Future studies should pay more attention to the effect of occupation physical activity among Chinese adults.

## Conclusions

This study found that a higher level of HPA and LTPA was associated with a lower risk of all-cause mortality, while no significant association was observed with commute activity. Our findings suggest people to perform HPA, especially LTPA, as a strategy for mortality reduction and health promotion. Future studies should pay more attention to the effect of occupational physical activity.

## Data Availability Statement

The raw data supporting the conclusions of this article will be made available by the authors, without undue reservation.

## Ethics Statement

The studies involving human participants were reviewed and approved by Ethical Review Committee for Biomedical Research, School of Public Health, Sun Yat-sen University. The patients/participants provided their written informed consent to participate in this study.

## Author Contributions

XL conceived and designed the study. PQ and YC collected the mortality data. HD, MZ, JH, and XL collected all other data. PH analyzed the data. PH, MZ, and JH drafted the manuscript. XL, HD, XZ, HW, and WZ reviewed and edited the manuscript. All co-authors provided comments and approved the final version.

## Funding

This work was supported by the Science and Technology Program of Guangzhou City (No. 202102080404), the Guangdong Basic and Applied Basic Research Foundation (No. 2022A1515010686), the National Natural Science Foundation of China (No. 72061137002), the Guangdong Provincial Key R&D Program (No. 2019B020230004), and the Medical Science and Technology Research Fund of Guangdong Province (No. A2021129). The founder had no role in the design, analysis, or writing of this manuscript.

## Conflict of Interest

The authors declare that the research was conducted in the absence of any commercial or financial relationships that could be construed as a potential conflict of interest.

## Publisher's Note

All claims expressed in this article are solely those of the authors and do not necessarily represent those of their affiliated organizations, or those of the publisher, the editors and the reviewers. Any product that may be evaluated in this article, or claim that may be made by its manufacturer, is not guaranteed or endorsed by the publisher.
