# Supplementary material for: Association of Habitual Physical Activity With the Risk of All-Cause Mortality Among Chinese Adults: A Prospective Cohort Study
Source: Front Public Health. 2022 Jun 24;10:919306. doi: 10.3389/fpubh.2022.919306 (PMC9263353; doi:10.3389/fpubh.2022.919306)
Supplement: Supplementary file 1 [file Data_Sheet_1.pdf]

Supplementary materials

Table S1. The characteristics of participants by habitual physical activity

Table S2. Sensitivity analysis on associations of all-cause mortality with habitual physical activity and leisure-time physical activity by using new cut-off points

Table S3. Sensitivity analysis on the associations of all-cause mortality with physical activity by excluding participants who died within the first year (N=11956)

Table S4. Sensitivity analysis on the associations of all-cause mortality with physical activity by excluding participants aged 80 years or more (N=11483)

Table S5. Sensitivity analysis on the associations of all-cause mortality with physical activity by excluding participants who died due to traffic accidents and injuries (N=11985)

Table S1. The characteristics of participants by habitual physical activity

Characteristic	Total (N=11994)	Low HPA (N=4020)	Moderate HPA (N=3976)	High HPA (N=3998)	*P* value
Age, years, mean (S.D.)	58.67 (11.78)	59.30 (12.94)	58.07 (11.76)	58.62 (10.49)	0.010*
Body mass index, kg/m^2, mean (S.D.)	23.98 (3.53)	24.05 (3.64)	24.01 (3.47)	23.87 (3.46)	0.022*
Sex, N (%)					<0.001†
Male	4194 (34.97)	1948 (48.46)	1316 (33.10)	930 (23.26)	
Female	7800 (65.03)	2072 (51.54)	2660 (66.90)	3068 (76.74)	
Education, N (%)					<0.001†
< high school	7518 (62.68)	2703 (67.24)	2498 (62.83)	2317 (57.95)	
High school	2914 (24.30)	855 (21.27)	942 (23.69)	1117 (27.94)	
> high school	1562 (13.02)	462 (11.49)	536 (13.48)	564 (14.11)	
Marital status, N (%)					0.159†
Married	10202 (85.06)	3385 (84.20)	3392 (85.31)	3425 (85.67)	
Others	1792 (14.94)	635 (15.80)	584 (14.69)	573 (14.33)	
Smoking					<0.001†
Never	9438 (78.69)	2790 (69.40)	3206 (80.63)	3442 (86.09)	
Former	718 (5.99)	288 (7.16)	228 (5.73)	202 (5.05)	
Current	1838 (15.32)	942 (23.43)	542 (13.63)	354 (8.85)	
Alcohol drinking					<0.001†
Never	9435 (78.66)	3032 (75.42)	3166 (79.63)	3237 (80.97)	
Occasionally	1835 (15.30)	623 (15.50)	615 (15.47)	597 (14.93)	
Frequent	724 (6.04)	365 (9.08)	195 (4.90)	164 (4.10)	
Hypertension					<0.001†
Yes	3796 (31.65)	1361 (33.86)	1253 (31.51)	1182 (29.56)	
No	8198 (68.35)	2659 (66.14)	2723 (68.49)	2816 (70.44)	
Dyslipidemia					0.538†
Yes	8426 (70.25)	2798 (69.60)	2809 (70.65)	2819 (70.51)	
No	3568 (29.75)	1222 (30.40)	1167 (29.35)	1179 (29.49)	
Diabetes					0.872†
Yes	982 (8.19)	332 (8.26)	330 (8.30)	320 (8.00)	
No	11012 (91.81)	3688 (91.74)	3646 (91.70)	3678 (92.00)	
COPD					0.037†
Yes	674 (5.62)	256 (6.37)	213 (5.36)	205 (5.13)	
No	11320 (94.38)	3764 (93.63)	3763 (94.64)	3793 (94.87)	
Cardiovascular disease					0.060†
Yes	599 (4.99)	227 (5.65)	190 (4.78)	182 (4.55)	
No	11395 (95.01)	3793 (94.35)	3786 (95.22)	3816 (95.45)	

Abbreviations: COPD, chronic obstructive pulmonary disease.

* *P* values of continuous variables were from one-way analysis of variance;

† *P* values of categorical variables were from chi-square tests.

Table S2. Sensitivity analysis on associations of all-cause mortality with Habitual physical activity and Leisure-time physical activity by using new cut-off points

	Deaths/Person-years	Crude HR (95% CI) †	Adjusted HR (95% CI) ‡	Adjusted HR (95% CI) §
**Habitual physical activity***				
≤10 MET-h/week	38/4039	1.00	1.00	/
10-50 MET-h/week	125/20659	0.64 (0.45, 0.92)	0.82 (0.57, 1.19)	/
>50 MET-h/week	45/13017	0.37 (0.24, 0.56)	0.61 (0.39, 0.95)	/
P for trend		<0.001	0.024	/
Every 10 MET-h/week increment		0.89 (0.85, 0.94)	0.94 (0.90, 0.99)	/
**Leisure-time physical activity***				
≤10 MET-h/week	41/5501	1.00	1.00	1.00
10-50 MET-h/week	125/20586	0.81 (0.57, 1.16)	0.84 (0.60, 1.21)	0.86 (0.60, 1.23)
>50 MET-h/week	42/11627	0.48 (0.31, 0.74)	0.62 (0.40, 0.96)	0.63 (0.41, 0.98)
P for trend		<0.001	0.038	0.048
Every 10 MET-h/week increment		0.91 (0.87, 0.96)	0.94 (0.90, 0.99)	0.94 (0.90, 0.99)

*Physical activity was categorized as ≤10 MET-h/week, 10-50 MET-h/week, >50 MET-h/week, corresponding to less than150 minutes per week, 150-750 minutes per week, and more than 750 minutes per week of moderate intensity physical activity.

† Crude HR, without any adjustment.

‡Adjustment for age, sex, education, marital status, smoking, alcohol drinking, body mass index, physician-diagnosed hypertension, diabetes, dyslipidemia, cardiovascular disease, and chronic obstructive pulmonary disease;

§Further adjustment for leisure-time physical activity (for commute activity) or commute activity (for leisure-time physical activity).

Table S3. Sensitivity analysis on the associations of all-cause mortality with physical activity by excluding participants who died within the first year (N=11956)

	Deaths/Person-years	Crude HR (95% CI) *	Adjusted HR (95% CI) †	Adjusted HR3 (95% CI) ‡
**Habitual physical activity**				
Low (≤ 24.5 MET-h/week)	83/12524	1.00	1.00	/
Moderate (24.5 - 51.1 MET-h/week)	52/12520	0.62 (0.44, 0.88)	0.84 (0.59, 1.20)	/
High (> 51.1 MET-h/week)	35/12650	0.41 (0.28, 0.62)	0.63 (0.42, 0.95)	/
P for trend		<0.001	0.027	/
Every 10 MET-h/week increment		0.89 (0.84, 0.94)	0.94 (0.89, 0.99)	/
**Leisure-time physical activity**				
Low (≤ 22.4 MET-h/week)	70/12826	1.00	1.00	1.00
Moderate (22.4 – 46.9 MET-h/week)	60/12377	0.88 (0.63, 1.25)	0.84 (0.59, 1.19)	0.84 (0.60, 1.19)
High (> 46.9 MET-h/week)	40/12490	0.58 (0.40, 0.86)	0.67 (0.45, 0.99)	0.68 (0.46, 1.00)
P for trend		0.006	0.047	0.062
Every 10 MET-h/week increment		0.90 (0.86, 0.96)	0.93 (0.89, 0.99)	0.94 (0.89, 0.99)
**Commute activity**				
Retirement (0 MET-h/week)	131/21192	1.00	1.00	1.00
Low (< 10 MET-h/week)	28/12467	0.37 (0.25, 0.55)	1.52 (0.97, 2.38)	1.41 (0.89, 2.22)
High (≥ 10 MET-h/week)	11/4034	0.44 (0.24, 0.81)	2.19 (1.12, 4.27)	2.00 (1.00, 3.93)
P for trend		0.002	0.016	0.038
Every 10 MET-h/week increment		0.58 (0.40, 0.85)	1.24 (0.97, 1.59)	1.21 (0.94, 1.56)

*Crude HR, without any adjustment.

†Adjustment for age, sex, education, marital status, smoking, alcohol drinking, body mass index, physician-diagnosed hypertension, diabetes, dyslipidemia, cardiovascular disease, and chronic obstructive pulmonary disease;

‡ Further adjustment for leisure-time physical activity (for commute activity) or commute activity (for leisure-time physical activity).

Table S4. Sensitivity analysis on the associations of all-cause mortality with physical activity by excluding participants aged 80 years or more (N=11483)

	Deaths/Person-years	Crude HR (95% CI) *	Adjusted HR (95% CI) †	Adjusted HR (95% CI) ‡
**Habitual physical activity**				
Low (≤ 24.5 MET-h/week)	71/11969	1.00	1.00	/
Moderate (24.5 - 51.1 MET-h/week)	51/12067	0.71 (0.50, 1.02)	0.88 (0.61, 1.27)	/
High (> 51.1 MET-h/week)	36/12137	0.50 (0.33, 0.74)	0.68 (0.45, 0.99)	/
P for trend		<0.001	0.047	/
Every 10 MET-h/week increment		0.91 (0.86, 0.96)	0.94 (0.89, 0.99)	/
**Leisure-time physical activity**				
Low (≤ 22.4 MET-h/week)	62/12209	1.00	1.00	1.00
Moderate (22.4 – 46.9 MET-h/week)	55/11837	0.91 (0.63, 1.31)	0.81 (0.56, 1.17)	0.81 (0.56, 1.17)
High (> 46.9 MET-h/week)	41/12128	0.66 (0.45, 0.98)	0.67 (0.45, 1.01)	0.68 (0.46, 1.03)
P for trend		0.040	0.058	0.068
Every 10 MET-h/week increment		0.93 (0.88, 0.98)	0.94 (0.89, 0.99)	0.94 (0.89, 0.99)
**Commute activity**				
Retirement (0 MET-h/week)	117/19755	1.00	1.00	1.00
Low (< 10 MET-h/week)	29/12402	0.40 (0.27, 0.60)	1.34 (0.85, 2.13)	1.26 (0.79, 2.01)
High (≥ 10 MET-h/week)	12/4017	0.50 (0.28, 0.91)	1.94 (1.01, 3.73)	1.79 (0.93, 3.48)
P for trend		0.007	0.046	0.084
Every 10 MET-h/week increment		0.66 (0.46, 0.94)	1.15 (0.91, 1.46)	1.13 (0.89, 1.44)

*Crude HR, without any adjustment.

†Adjustment for age, sex, education, marital status, smoking, alcohol drinking, body mass index, physician-diagnosed hypertension, diabetes, dyslipidemia, cardiovascular disease, and chronic obstructive pulmonary disease;

‡Further adjustment for leisure-time physical activity (for commute activity) or commute activity (for leisure-time physical activity).

Table S5. Sensitivity analysis on the associations of all-cause mortality with physical activity by excluding participants who died due to traffic accidents and injuries (N=11985)

	Deaths/Person-years	Crude HR (95% CI) *	Adjusted HR (95% CI) †	Adjusted HR (95% CI) ‡
**Habitual physical activity**				
Low (≤ 24.5 MET-h/week)	96/12524	1.00	1.00	/
Moderate (24.5 - 51.1 MET-h/week)	60/12514	0.62 (0.45, 0.86)	0.86 (0.62, 1.19)	/
High (> 51.1 MET-h/week)	43/12662	0.44 (0.31, 0.63)	0.68 (0.47, 0.99)	/
P for trend		<0.001	0.046	/
Every 10 MET-h/week increment		0.89 (0.85, 0.94)	0.95 (0.90, 0.99)	/
**Leisure-time physical activity**				
Low (≤ 22.4 MET-h/week)	82/12826	1.00	1.00	1.00
Moderate (22.4 – 46.9 MET-h/week)	68/12378	0.86 (0.62, 1.18)	0.83 (0.60, 1.15)	0.83 (0.60, 1.15)
High (> 46.9 MET-h/week)	49/12496	0.61 (0.43, 0.87)	0.72 (0.50, 1.03)	0.72 (0.51, 1.04)
P for trend		0.006	0.070	0.084
Every 10 MET-h/week increment		0.92 (0.87, 0.96)	0.94 (0.90, 0.99)	0.95 (0.90, 0.99)
**Commute activity**				
Retirement (0 MET-h/week)	157/21203	1.00	1.00	1.00
Low (< 10 MET-h/week)	31/12465	0.34 (0.23, 0.50)	1.31 (0.85, 2.00)	1.22 (0.79, 1.88)
High (≥ 10 MET-h/week)	11/4032	0.37 (0.20, 0.68)	1.63 (0.85, 3.14)	1.50 (0.77, 2.91)
P for trend		<0.001	0.121	0.213
Every 10 MET-h/week increment		0.52 (0.36, 0.76)	1.15 (0.90, 1.47)	1.12 (0.86, 1.45)

*Crude HR, without any adjustment.

†Adjustment for age, sex, education, marital status, smoking, alcohol drinking, body mass index, physician-diagnosed hypertension, diabetes, dyslipidemia, cardiovascular disease, and chronic obstructive pulmonary disease;

‡ Further adjustment for leisure-time physical activity (for commute activity) or commute activity (for leisure-time physical activity).
